# Scratch Behaviour of Bulk Silicon Nitride Ceramics

**DOI:** 10.3390/mi12060707

**Published:** 2021-06-16

**Authors:** Xiaolan Xiao, Jiayun Deng, Qiang Xiong, Qiusheng Yan, Zhengtao Wu, Huatay Lin

**Affiliations:** School of Mechanical and Electrical Engineering, Guangdong University of Technology, Guangzhou 510006, China; xxlan@gdut.edu.cn (X.X.); dengjiayun@mail2.gdut.edu.cn (J.D.); xiongqiang@gdut.edu.cn (Q.X.); ztwu@gdut.edu.cn (Z.W.); huataylin@comcast.net (H.L.)

**Keywords:** Si_3_N_4_ ceramics, scratch, indentation, mechanical removal

## Abstract

Si_3_N_4_ ceramic is generally recognized as being difficult to machine due to its hardness and brittleness. It is necessary to control the normal load applied and the machined depth of the abrasive particles in order to eliminate surface/subsurface damage and defects during the grinding or polishing. In this study, scratch experiments were conducted on the polished surface of Si_3_N_4_ specimens to investigate the brittle–ductile transformation and the evolution of material removal mechanisms. In addition, the cracking behaviour of Si_3_N_4_ ceramic was characterized by indentation tests. The Vickers indentation produced cracks that exhibited good developmental integrity and geometric symmetry. The results indicate that the scratch track can be divided into three stages: the ductile regime, the brittle–ductile coexisting stage, and the brittle fracture regime. The critical loads and the corresponding penetration depths of cracking occurrence in Si_3_N_4_ were recorded. The material removal of Si_3_N_4_ ceramic was primary attributed to ductile regime removal when the load was less than 9.8 N. Microcrack initiation on the subsurface was observed when the penetration depth of the scratch tip reached 8 μm or the depth of the indentation tip reached 3.2 μm. Microcracks expanded rapidly as the load was further increased, resulting in a brittle fracture of the Si_3_N_4_ ceramic.

## 1. Introduction

Silicon nitride (Si_3_N_4_) ceramics are widely used in various industrial sectors owing to their excellent physical–mechanical properties, such as their high strength, low density, high thermal shock resistance, low friction coefficient, high abrasion, and corrosion resistance. However, the hardness and brittleness make parts made of Si_3_N_4_ ceramics difficult to machine [1,2]. In general, surface defects, including scratches, dimples, pit defects, and microcracks, can be found on the surface after abrasive polishing due to the fracture of the ceramic structure. This significantly impacts the quality of the surface finishing and the performance of parts. Extensive studies concerning the deformation and fracture behaviours of Si_3_N_4_ ceramics provide valuable information about the anticipated deformation systems, plasticity onset, and incipient fracture patterns [3,4,5,6,7,8].

During the polishing of Si_3_N_4_ ceramics, the material removal process consists of ductile deformation, brittle fracture, and powdering of the superficial material [9]. Generally, the machining efficiency is higher when the material removal mechanism is fracture dominated [10]; however, the formation of microfractures and dimples on the machined surface is inevitable. In contrast, the number and severity of fracture-induced defects are reduced when ductile deformation dominates the material removal process [11]. If the thickness of the chipping materials is below a critical value, the material can be removed under a ductile model along with the chipping materials to generate a crack-free surface [12]. Furthermore, Liu et al. [13] reported the effect of the process parameters on the ground surface quality and grinding forces during diamond grinding of silicon nitride. It was demonstrated that a low–depth cut leads to a smooth surface with enhanced strength, and vice versa. Aside from the critical cutting depth, it was reported that cutting parameters including external load, the feed rate to the grinder, and the size of the abrasive grains also played critical roles in the material removal mechanisms in the polishing of Si_3_N_4_ ceramic surfaces [14,15,16]. Similar results were reported by Xiao et al. [17], wherein surface defects became more severe with the increment of abrasive force induced by the applied load and the size of abrasive particles. It was reported that the abrasive force was the main factor affecting the depth of damage beneath the machined surface. Specifically, the microhardness of Si_3_N_4_ ceramic was changed when the surface was polished with a larger externally applied load, which caused an increase in the thickness of the subsurface damage. Nazir et al. [18] developed a mathematical model to investigate the development of cracks in the subsurface region. The results showed that the development of cracks and fractures was ascribed to the fatigue of facial materials during the abrasion process. The propagation uncertainty of cracks was evaluated using surrogate models built on highly accurate finite element modelling for equivalent stress intensity factors. Image processing [19] combined with transmission electron microscopy (TEM) analysis [20] was used to examine the thickness of the subsurface damage layer on the polished and etched surface of ceramic materials. Bobzin et al. [21] investigated the mechanism of ductile deformation using TEM analyses. Transmission electron microscopy and scanning transmission electron microscopy (STEM) were applied to explore the mechanism of ductile deformation of ceramic materials.

In order to further investigate the material removal mechanism of Si_3_N_4_ ceramics based on single grinding particle cutting, single grinding particle cutting tests were integrated into the grinding area. Thereafter, various physical phenomena during the grinding process were evaluated and reported [22]. Theoretical research was conducted to investigate the material removal process in the polishing of ceramic materials. The molecular dynamics method [23] and finite element method [24] were employed to simulate the deformation mechanisms during the material removal process of ceramics. Liu [25,26] et al. conducted the simulation and experiment of cutting Si_3_N_4_ ceramics using a single diamond abrasive approach and concluded that the material removal of Si_3_N_4_ ceramics is mainly attributed to microductile deformation combined with little bits of brittle fracture. On the other hand, Zhang et al. [27] investigated the relationship between the applied normal load and cutting depth. The influences of elastic recovery and stress distribution of materials were considered in the theoretical model developed. The results showed that the effects of elastic recovery of the material, the geometry of the tool tip, and the stress distribution at the interface between the tip and sample significantly influenced the machined depth. Wang et al. [28] investigated the mechanism of ductile deformation and crack initiation/evolution in machining sapphire. The results showed that the material removal exhibited three stages depending on the scratching depth: the ductile stage at a short depth (less than 0.73 μm), characterized by a smooth scratching groove, curled shavings, and tiny stick-slip lines; ductile–brittle fractures coexisted in the second stage, in which the initial radial cracks, tearing, and segmental chips appeared; brittle fractures dominated the final stage, with irregular debris and spalling occurring due to the intersection of lateral and radial cracks.

Therefore, the normal load and machined depth should be carefully selected in the polishing of Si_3_N_4_ ceramics, because the material removal process should be a ductile-dominated mode in order to obtain a relatively intact surface finish. In this work, to obtain the critical load and critical machined depth at which the plasticity/brittleness removal transition occurred when Si_3_N_4_ ceramics were processed under normal temperature and pressure conditions, the microscopic strain and damage on the surface of Si_3_N_4_ ceramic material under the action of abrasive particles were studied by scratch and indentation experiments using diamond tips. The mechanism of microcrack propagation and brittle/ductile regime removal transitions are discussed according to the experimental results. This could be used to identify different material removal stages to improve the machining and polishing of Si_3_N_4_ ceramic components manufactured for different industrial applications.

## 2. Materials and Experiments

### 2.1. Materials

**Figure 1:**
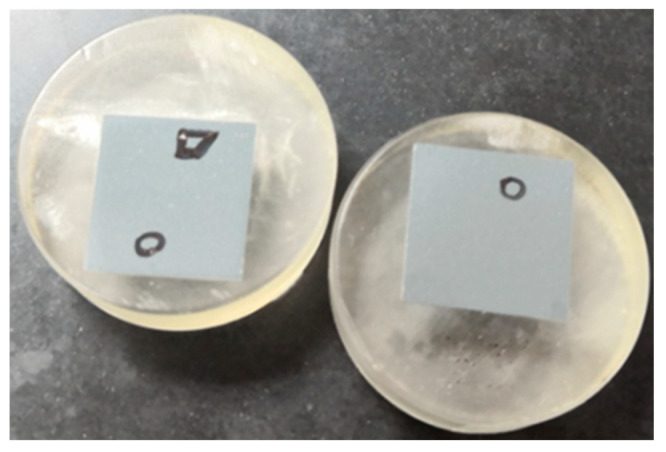
Photos of polished surfaces of silicon nitride ceramic specimens prepared for scratching and indentation experiments.

Si_3_N_4_ ceramic workpieces processed by hot isostatic pressing (acquired from Shenzhen Hard Precision Ceramic Co., Ltd., Shenzhen, China) were used in this study. Their properties are listed in Table 1. The Si_3_N_4_ workpieces were polished with a mirror surface finish to eliminate the influence of surface roughness and flatness on the experimental results. As shown in Figure 1, the Si_3_N_4_ workpieces were embedded in resin blocks, and the bottoms of the resin blocks were polished into flat faces. The top surfaces of the Si_3_N_4_ ceramics were polished to remove the craters, cracks, and pores caused by the specimen cutting process. The surface profile information of the samples after polishing is listed in Table 2.

### 2.2. Scratch Experiments

**Figure 2:**
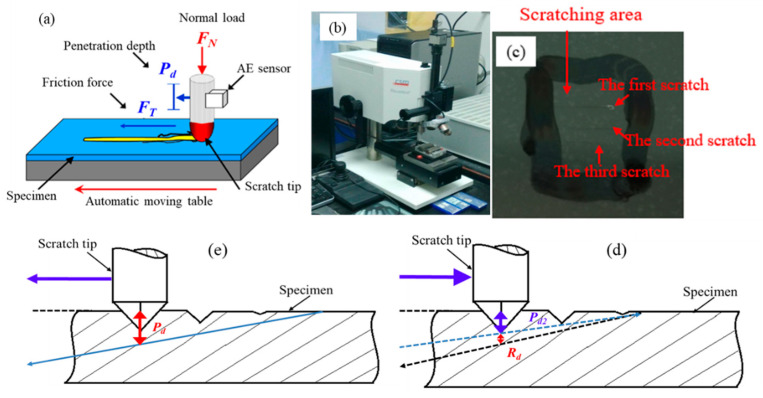
Experimental method: (**a**) schematic; (**b**) scratching experiment setup (CSM Revetest Scratch Tester); (**c**) scratching area; (**d**) schematic diagram of the curve *P_d_* (the penetration depth of the tip); (**e**) schematic diagram of the curve *R_d_* (the difference between *P_d_* and residual depth of the scratch track) and mean elastic recovery.

The scratch experiments were conducted to investigate the interaction between the Si_3_N_4_ ceramic surface and abrasive particles in the moving state under a linear load of *F_N_*. During the experiment, a hard tip scratched across the workpiece surface with a linearly increased load of *F_N_*. By monitoring changes in the *P_d_* curve (the penetration depth of the tip) and *R_d_* (obtained by subtracting the residual depth of the scratch track from *P_d_*), the scratch morphology and microcrack propagation could be investigated in real time, as shown in Figure 2. When the brittle fractures (microcracks) appeared, both the critical load *F_N_* and the critical penetration depth *P_d_* were recorded, which were used to evaluate the brittle–ductile transition of the workpiece.

In this study, a Revetest^®^ scratch test system (CSM Instruments, Peseux, Switzerland) with a Rockwell diamond tip (Ø 200 μm) was used to conduct the scratching tests, as shown in Figure 2b. During the scratching experiment, the scratch tip traversed the surface of the Si_3_N_4_ ceramic sample at a speed of 6 mm/min, and the scratch length was 3 mm. The normal indenter load applied was increased from 0.9 N to 50 N with a constant loading rate of 98.20 N/min.

Three sets of scratch tracks were produced on the sample, and the average values of critical load *F_n_* and critical penetration depth *P_d_* were calculated for the workpiece. Before the scratching experiments, the surface of each sample was scanned using the scratch tip under a low loading 0.9 N to scan the surface undulation height and roughness of the moving sample. The surface profiles showed that height differences (based on the profile curve *P_f_*) were less than 1 μm within the 0–3 mm field of view. Notwithstanding, the surface profiles were relatively smooth, indicating an evenly flat surface suitable for valid scratching experiments.

### 2.3. Indentation Experiments

To further investigate the formation and propagation mechanism of microcracks, indentation experiments were conducted. Because the Vickers indentation crack exhibited good developmental integrity and geometric symmetry, the shape of the indentation head was similar to the diamond grinding particle used in the experiment.

Previous research [30] showed that the applied overload could lead to the initiation and growth of microcracks, and eventually to the formation of large visible cracks on the material’s surface when the internal stress exceeded the minimum critical failure stress. According to Griffith’s brittle-fracture crack growth criterion, combined with the energy balance principle, the critical failure stress is given by Formula (1) [31].

(1) $$\sigma_{cr}=\left(\frac{2\gamma E}{{\pi c}_{r}}\right)$$

where *σ_cr_, C_r_, γ,* and *E* are the critical failure stress, half-length of the crack, free energy per unit surface area, and elastic modulus, respectively.

**Figure 3:**
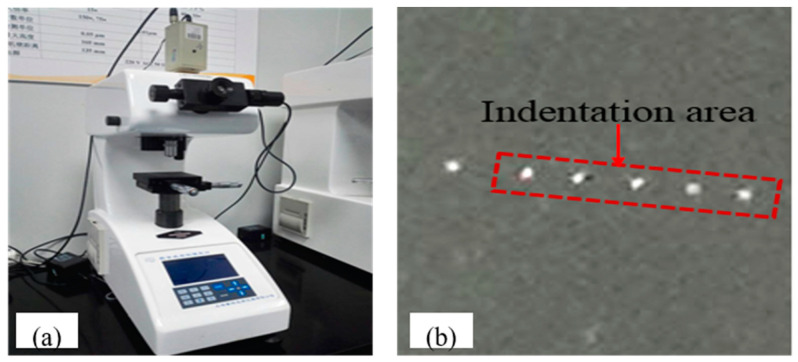
Indentation experiments: (**a**) experiment setup (HXD-2000TM /LCD microhardness tester); (**b**) specimens after indentation experiments.

In this study, Vickers indentation tests were performed on an automatic turret digital display microhardness tester (HXD-2000TM/LCD, Chengdu Yingdu Technology Co., Ltd., Chengdu, China), as shown in Figure 3a. The tester was equipped with an image acquisition and signal control system, which presented real-time loading, observations, and measurements via the computer software, as shown in Figure 3b. Indentation loads were 1.96 N, 2.94 N, 4.9 N, 9.8 N, and 19.6 N, and the constant holding load time was 10 s.

## 3. Results and Discussion

### 3.1. Analysis of Scratching Experiments

**Figure 4:**
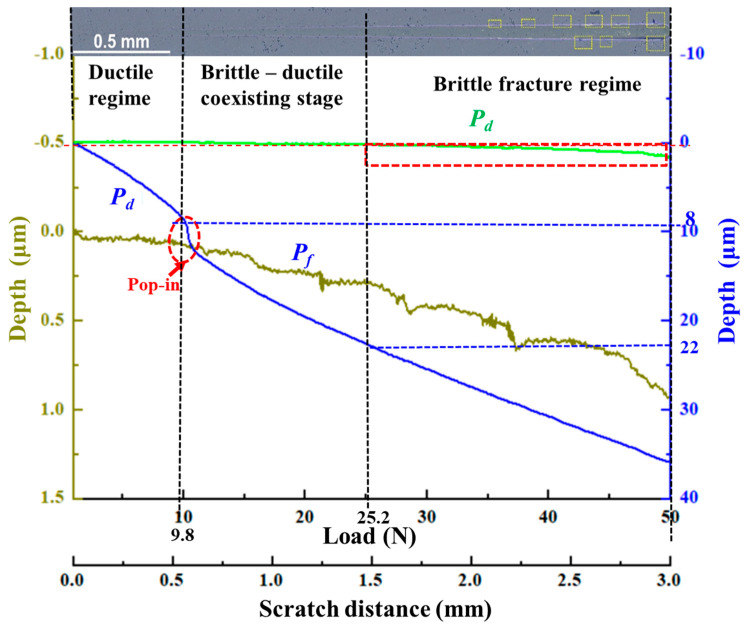
The *P_d_*, *R_d_*, and *P_f_* curves in the scratching experiment (1–50 N loading).

Figure 4 presents the changes in the curves of *P_d_* and *R_d_* as a function of the scratch length with the movement of the diamond scratch tip. Specifically, a “pop-in” phenomenon caused by microcracking was observed in the *P_d_* curve at the load of 9.8 N along with the scratch. After the pop-in position, the material removal process changed from ductile removal to brittle removal with the indenter’s movement. This transition was attributed to the fact that the scratch tip rapidly penetrated the surface of the Si_3_N_4_ ceramic material when pop-in occurred, resulting in the initiation and propagation of microcracks in the ceramics [32,33,34]. Compared with the linear increase in the *P_d_* curve, the *R_d_* curve remained relatively unchanged from 0 to 25.2 N under these study conditions. The difference between the recorded scratch depth (as shown by the dotted red line that coincides with the *R_d_* curve) during loading and the scanned scratch depth value after unloading (as shown by the solid green line) was very insignificant, suggesting that the ductile deformation mode dominated the material removal process in this region. When the load reached 25.2 N, the curve *R_d_* gradually exhibited a downward trend, as shown inside the red dashed box. Plenty of cracks and shedding of granular material could be observed. Moreover, brittle fracture marks clearly appeared on both sides of the scratch. The apparently elastic recovery depth was pronounced in the ceramic specimens, and the initiation and growth of large cracks occurred in this region.

**Figure 5:**
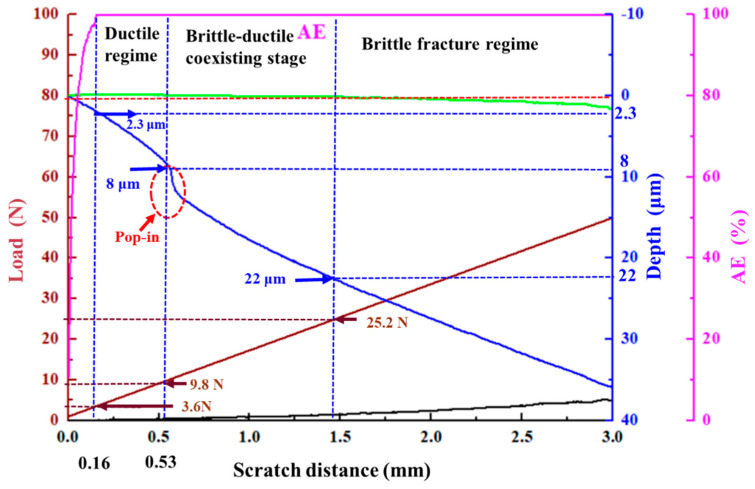
Main load-displacement curves of the scratching experiment (1–50 N loading).

The acoustic emission (AE) signal was used to monitor the microscale material deformation in this study. The AE signal strength values are known to be in proportion to the material removal rate, and their fluctuation is correlated to the random fracture. The AE signals obtained by wavelet packet decomposition could be applied to identify the different material removal stages [28]. The results showed that the AE signal varied, increasing linearly with the load during the ductile deformation process, as shown in Figure 5. The ductile deformation mode dominated the removal process until the pop-in phenomenon occurred, which meant either a change in the state of the material or the initiation of microcracks. It was assumed that the brittle removal mode gradually took over as the dominant material removal process with the increase in the applied normal load.

Combined with the corresponding load-displacement curves, it was observed that the microscale material removal deformation mode changed when the pop-in occurred in the *P_d_* curve, in which the load was 9.8 N, and the critical depth of the cut was 8 μm at this position. When the load reached 3.6 N, and the machined depth was 2.3 μm, the AE signal strength reached its upper limit, and then, with the linear loading, the formation of microstrains and fractures became more prominent until the microcracks occurred at the pop-in stage. When the load reached 25.2 N, the *R_d_* curve exhibited a downward trend, and this elastic recovery suggested that a large number of cracks and shedding of granular material occurred. The initiation and growth of large cracks implied that this region had transitioned into a brittle fracture regime.

According to the *P_d_*, *R_d_*, and AE curve analyses, the scratch region could be divided into three stages: the ductile regime, the brittle–ductile coexisting stage, and the brittle fracture regime. A load of 9.8 N was defined as the critical value of ductile–brittle removal transformation because the pop-in occurred, and the microcrack initiation and growth was detected and observed. The material removal process then transitioned into the coexistence of ductile deformation and brittle fracture. Elastic deformation existed throughout the whole process, but after 25.2 N, the elastic recovery was more apparent, suggesting that a large number of brittle fractures occurred.

**Figure 6:**
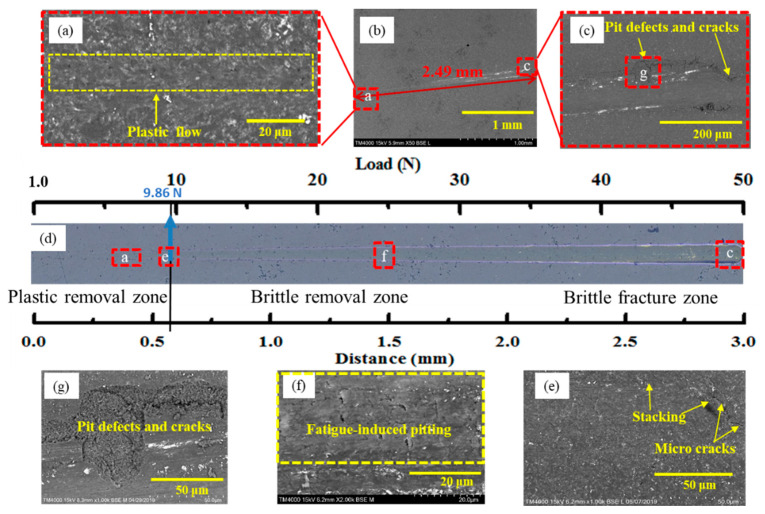
SEM micrographs of surface morphology after scratching under an applied load of 1–50 N: (**a**) full view; (**b**) ductile regime; (**c**) brittle–ductile coexisting stage; (**d**) brittle fracture regime; (**e**) morphology of pit defects; (**f**) morphology of fatigue-induced pitting; (**g**) morphology of pit defects and cracks.

Figure 6 shows the micromorphology of the 0–3 mm scratch length at different magnifications. A specific area of each region was selected for SEM observation, as shown in the small red boxes in Figure 6a. Scratch morphologies within the small area were analysed using SEM, as shown in Figure 6b–e. Figure 6b showed the surface morphology of the materials before the initiation of the pop-in event. The observations showed features of ductile flow deformation with no formation of microcracks on the surface of the test sample. To more clearly observe the surface morphology of the brittle–ductile coexisting stage, a small area with prominent markings near 1.5 mm from the left end of the scratch was selected for further detailed examination, as shown in Figure 6c. Ductile deformation and brittle fracture coexisted in the second stage, where the pits, tearing, initial radial cracks, and chips appeared. In addition, the brittle fracture regime was visible to the right of the scratch traces, as shown in Figure 6d, and a large number of cracks, pits, and spalling could be observed as well. The material on both sides of the scratch was stacked in a granular shape, and big cracks were visible (Figure 6g). The surface morphology of the pit defects was further examined, as shown in Figure 6e. Brittle fractures dominated the third stage, and irregular pits and spalling occurred due to the big cracks (Figure 6f). The material in the pit defects was dispersed in a granular pattern, which would seriously affect the properties and performance of the material. Therefore, the formation of pits and cracks should be avoided during processing.

**Figure 7:**
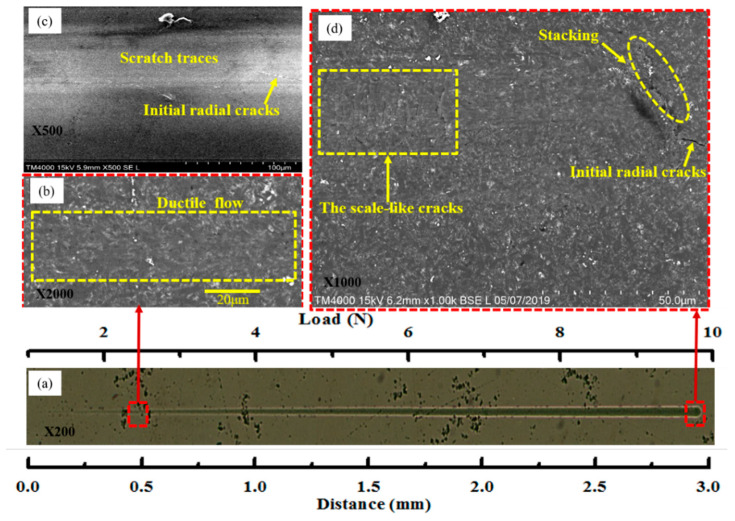
Surface morphology of silicon nitride after scratching (SEM) at a 1–10 N loading: (**a**) full view; (**b**) ductile removal stage; (**c**) brittle removal stage; (**d**) morphology of brittle removal stage.

Furthermore, additional scratching tests were performed to investigate the characteristics of the ductile deformation process of Si_3_N_4_ ceramics under applied normal loads of less than 10 N. Figure 7 shows the SEM micrographs of the scratch morphologies on the Si_3_N_4_ surface under the load ranging from 1.0 N to 10 N.

**Figure 8:**
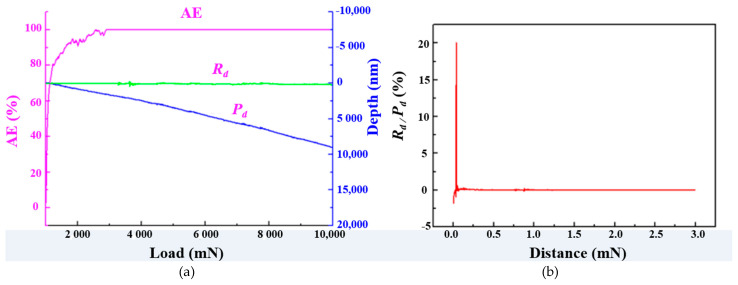
Results of scratching experiment (1–10 N loading): (**a**) main load–displacement curves; (**b**) elastic deformation shape.

Figure 7a presents a full view of the scratch path. According to the previous analysis, when the load is less than 9.8 N, no microcracks appear on the Si_3_N_4_ ceramic surface, and the entire area undergoes ductile deformation (Figure 5). Figure 7b shows the area’s morphology close to the left-most end of the scratch at about 0.5 mm. The morphological characteristics of Si_3_N_4_ ceramics during elastic ductile deformation were clearly observed. The material exhibited regular ductile flow with no microcracks, pits, or stacking defects. Figure 7c presents a full view of the area’s morphology close to the right-most end of the scratch traces. The initial radial cracks of the surface material could be clearly observed in this region. When the load reached about 9.8 N, both scale-like cracks and radial cracks appeared at the right-most end of the scratch, and stacking of the surface material could also be clearly observed in this region, as shown in Figure 7d. The load-displacement curve for an applied load ranging 1.0–10 N is shown in Figure 8a, and the corresponding elastic deformation is shown in Figure 8b.

The *P_d_* curve does not show characteristics of sharp penetration of the scratch tip before pop-in appears, suggesting that there was no change in the material state under 1.0–10 N loading conditions, as shown in Figure 8a. Moreover, apart from a sizeable amount of elastic deformation at initial contact with the tip, elastic deformation was almost negligible throughout the entire scratch process, and ductile deformation was the dominant deformation process, as shown in Figure 8b.

Furthermore, the experiment illustrated that the diamond tip rapidly penetrated the surface of the Si_3_N_4_ ceramic material, leading to the initiation, growth, and propagation of microcracks in the ceramics when pop-in occurred in the *P_d_* curve, as shown in Figure 4. At this pop-in moment, the normal load was 9.8 N, and the machined depth was 8 μm. After the pop-in event, the material removal of the Si_3_N_4_ ceramic surface transitioned from ductile to brittle.

### 3.2. Results of the Indentation Experiments

As a result of the influence of the indenter and loading method, the scratching tests could only be employed to characterize the starting point and range of the propagation of a crack. Therefore, the mechanism of crack initiation/evolution in Si_3_N_4_ ceramics was evaluated by indentation tests to further investigate the crack propagation’s regularity.

**Figure 9:**
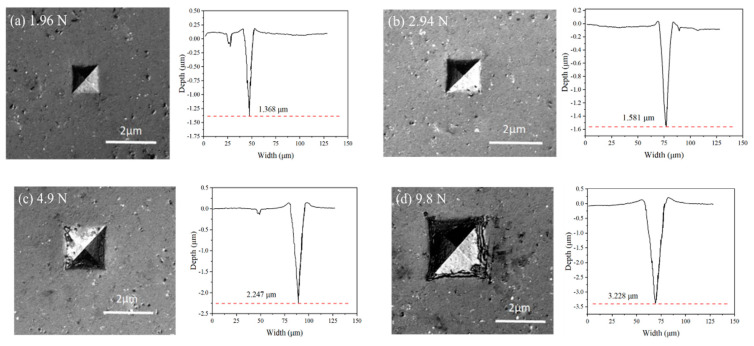

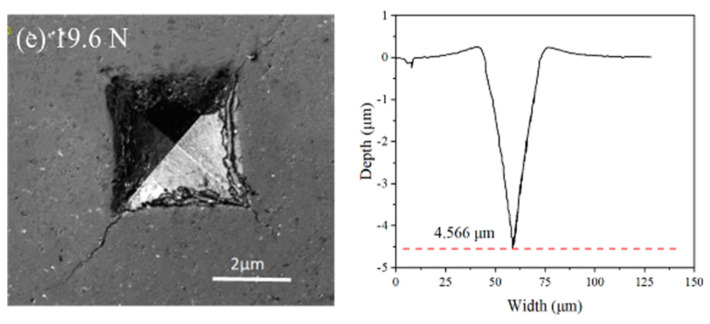
Indentation micrograph and depth curve of Si_3_N_4_ material under different loadings: (**a**) 1.96 N; (**b**) 2.94 N; **(c**) 4.9 N; (**d**) 9.8 N; (**e**) 19.6 N.

The test samples were examined under a laser confocal microscope (OLS4000, Olympus corporation, Tokyo, Japan) to better observe the Si_3_N_4_ surface morphology after indentation tests. Figure 9 shows the morphology and profiles of the indentations at different external loads: 1.96 N, 2.94 N, 4.9 N, 9.8 N, and 19.6 N. The observations show that no significant cracks were generated on the surface of samples when the externally applied loads were smaller than 19.8 N. The edge of the indentation was found to be collapsed along with multiple cracks when the force was larger than 9.8 N, as shown in Figure 9d. This was attributed to the initial microcrack generation that occurred in this region, and the phenomenon corresponded to the pop-in stage, as described in Figure 3. When the load increased to 19.8 N, large cracks were clearly observed on the surface, as shown in Figure 9e. Moreover, the extended cracks appeared at the opposite corner of the indentation. This was related to the fact that brittle fractures dominated this stage; a large number of cracks, pits, and spalling occurred; and the material on each side of the indenter was stacked in a granular shape.

**Figure 10:**
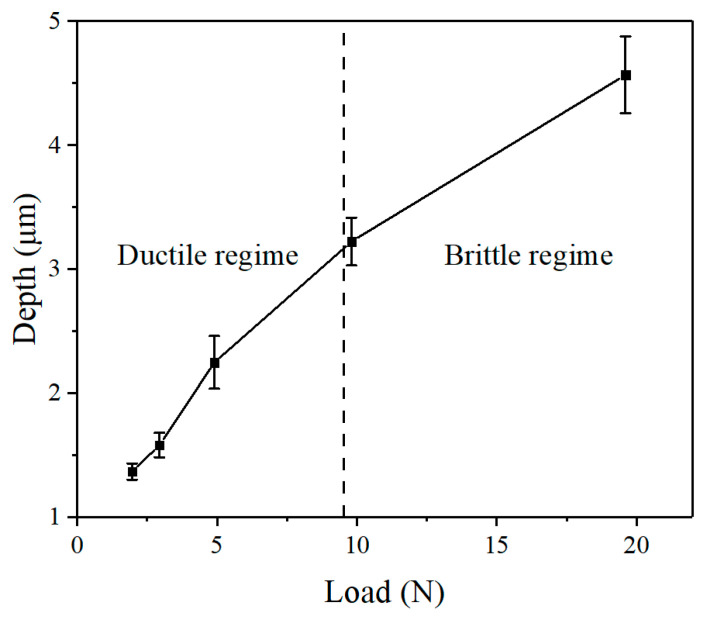
Relationship between the indentation depth and load.

Moreover, the measurement results show that the indentation depth increased with the increment of the external loads, as shown in Figure 10. There was a linear relationship between load and indentation depth during the ductile regime. When brittle fractures occurred (after 9.8 N), the rate of increase in indentation depth gradually shifted to a shorter depth, probably owing to the partial force decomposition caused by crack initiation and propagation. The results of the microscopy analysis obtained from the indentation tests were consistent with the scratch tests, which clearly identified the material removal modes under the various applied normal loads. Understanding the material removal mechanisms will help improve the machining and polishing of Si_3_N_4_ ceramic components manufactured for different industrial applications.

## 4. Conclusions

The scratching and indentation tests were conducted on a polished Si_3_N_4_ ceramic surface to investigate the material removal mechanisms, the critical normal load, and the machined depth as related to the occurrence of microcracks in the material. On the basis of the results obtained, the conclusions can be listed as follows:

1. Material removal exhibits three stages under increasing scratching depths: the ductile regime, the brittle–ductile coexisting stage, and the brittle fracture regime.
2. When the load is less than 9.8 N, the Si_3_N_4_ ceramic material is mainly removed via the ductile material removal process. When the load reaches ~9.8 N, the occurrence of the pop-in event suggests that microcrack initiation and growth have occurred on the subsurface of the Si_3_N_4_ sample, and the material removal process transitions from ductile removal to brittle removal.
3. The results show that 9.8 N is the critical value of ductile–brittle removal transformation because of the pop-in event, the critical machined depth of scratching is 8 μm, and the depth of indentation is 3.2 μm under the test conditions employed in this study. Moreover, the critical normal loads and machined depths from our experiments could be applied to identify the different material removal stages in the machining process.

## Figures and Tables

**Table 1:** The physical properties of Si_3_N_4_. Data from [29].

Density	Hardness	Elastic Modulus	Toughness	Flexure Strength
3.22 g/cm^3^	18 GPa	380 GPa	10.5 MPa·m^1/2^	550 MPa

**Table 2:** The dimensions of Si_3_N_4_ specimens for scratching and indentation tests.

Length	Width	Thickness	Surface Roughness (Ra)	Surface Roughness After Polishing (Ra)
15 mm	15 mm	0.8 mm	404 nm	80 nm
